# The impact of extreme summer temperatures in the United Kingdom on infant sleep: Implications for learning and development

**DOI:** 10.1038/s41598-023-37111-2

**Published:** 2023-06-21

**Authors:** Sarah E. Berger, Monica R. Ordway, Emiel Schoneveld, Maristella Lucchini, Shambhavi Thakur, Thomas Anders, Liza Natale, Natalie Barnett

**Affiliations:** 1 College of Staten Island and the Graduate Center of the City University New York, 2800 Victory Blvd., 4S-108, Staten Island, NY 10314 USA; 2 Yale School of Nursing, Orange, USA; 3 Yale School of Medicine, West Haven, USA; 4 Research Institute of Child Development and Education, University of Amsterdam, Amsterdam, Netherlands; 5 Nanit Lab, New York, USA; 6 Brown University, Providence, USA; 7 New York University Grossman School of Medicine, New York, USA

**Keywords:** Paediatric research, Sleep

## Abstract

The U.S. Global Change Research Program reports that the frequency and intensity of extreme heat are increasing globally. Studies of the impact of climate change on child health often exclude sleep, despite its importance for healthy growth and development. To address this gap in the literature, we studied the impact of unusually high temperatures in the summer of 2022 on infants’ sleep. Sleep was assessed objectively using Nanit camera monitors in infants’ homes. Generally, sleep was not impacted when temperatures stayed below 88° but was negatively impacted when temperatures reached over 100°. Compared to non-heatwave nights, infants had less total sleep, less efficient sleep, took longer to fall asleep, had more fragmented sleep, and parents’ visits were more frequent during the night. Following peaks in temperature, sleep metrics rebounded to better than average compared to non-peak nights, suggesting that infants compensated for disrupted sleep by sleeping more and with fewer interruptions once the temperature dropped below 85°. Increased instances of disrupted sleep in infancy have important implications for psychological health and development. Climate disruptions such as heat waves that create occasional or ongoing sleep disruptions can leave infants vulnerable and unprepared for learning.

## Introduction

Just as one requires a certain amount of water each day, so too does one require sleep for ongoing health^1^.

The U.S. Global Change Research Program (USGCRP) recently reported with “very high confidence” that the frequency and intensity of extreme heat events are increasing globally^2^. The average temperatures in recent decades have been much higher than at any other documented time. In fact, in 2022, Europe was identified as a “heatwave hotspot” with instances of extreme heat increasing continuously since the 2003 heatwave that was responsible for over 70,000 deaths^3^. The headline-grabbing portrayal of the impact of climate change on health typically features cataclysmic weather events stemming from temperature change, such as wildfires or hurricanes, that suddenly and directly cause injury or loss of life.

Yet increases in temperature also influence adults’ and adolescents’ physical and mental health in insidious, chronic ways. Increasing temperatures are associated with increased respiratory problems and allergies brought on by air pollution^4^; with aggression, anxiety, and depression^5,6^; and with disruption of sleep^1,7^. For example, in a comprehensive study of the relation between ambient temperature and sleep in human adults, Minor and colleagues documented over 10 billion minute-level sleep observations over 2 years using sleep-tracking wristbands worn by respondents^8^. Increased temperatures resulted in sleep loss, primarily because the onset of sleep was delayed. Moreover, the effects of increased temperatures on sleep were worse for older adults, females, and lower-income populations.

Several studies have shown that children are even more vulnerable to extreme heat than adults. Direct effects include heatstroke or asthma, increased risk for pre-term birth, and posttraumatic stress or attachment disorders associated with displacement after climate disasters. Indirect effects include psychological distress and malnutrition^5,9^. Young people experience more psychological distress about climate change than do adult^10^. A recent literature review revealed that the increase in air pollution associated with climate change elicited respiratory problems in children, which, in turn, contributed to decrements in sleep quality^11^.

Sleep is fundamental to healthy growth and development. Getting less than the recommended amount of nightly sleep in infancy increases the likelihood of being overweight in preschool, is associated with emotional and behavioral problems in early childhood, and is positively associated with poorer language development and problem-solving skills^12^. It is surprising, therefore, that scoping reviews on the impacts of climate change on child health often exclude sleep health^9^, especially given the well-established connection between climate and sleep quality. An age-matched comparison of infants’ sleep in the winter versus the summer demonstrated that circadian and ultradian cycles develop differently, according to exposure to temperature and light–dark cycles^13^. Infants’ sleep during the summer was characterized by later sleep time and more motor activity during sleep. Consistent, predictable exposure to environmental factors such as temperature over the first 6 months of life shaped typically developing sleep patterns in infancy. Yet other sleep metrics such as sleep efficiency, sleep duration, and number of wake episodes did not vary according to season. The connection between rising temperatures on children’s sleep is beginning to be established, but pediatric studies are few^14^. Specifically, it remains unclear whether sudden, unexpected changes in temperature, such as during heat waves, are experienced as a jolt to the system.

The scientific evidence linking the effects of climate change to disrupted sleep in young children is insufficient^1^. The predicted impact of climate change is an increase in acute weather events, yet we know very little about what happens to infants’ sleep in moments of extreme weather such as the 2022 heatwaves central to this study. Research into the effect of global temperature rising in vulnerable populations shows that above average temperatures reduce sleep quality^8^. However, the effect of extreme temperatures on infant sleep is unknown. We aim to address this gap in the literature by studying the impact of extreme heat in the summer of 2022 in the United Kingdom (UK) on infants’ and toddlers’ sleep. We expected infant sleep characteristics previously linked to environmental factors, such as sleep onset times and increased activity during sleep, as well as related behaviors, such as sleep efficiency, night wakings, and parental visits, to be negatively affected by high temperatures. We also asked whether heatwaves per se, defined as two or more consecutive days when the temperature exceeded the 85th percentile of historical temperatures for that location^15^, would disrupt infants’ sleep separately from the absolute value of the temperature, given their rarity and their extent over multiple days. Note that heatwaves are defined as relative to what is historical and that high temperatures are not the sole criteria, as illustrated in Fig. S1 where the heatwave in June, for example, is of similar temperatures as non-heatwave temperatures during July.

## Methods

### Participants

Parents of 413 infants (51% girls, 37% boys, 12% not reported; Mean age as of June 1, 2022 = 12.22 months, Median age = 11 months, SD = 5.48 months, Range = 2 months to 33 month) participated in this study. Participants resided in the greater London area of the UK. This location was chosen because high temperatures are unusual and because it is rare for homes to have air conditioning.

### Procedure

Users of the Nanit baby monitor received an email invitation to participate in a study investigating the association between sleep and climate change. Users who agreed to participate in the study gave their informed consent to share objective sleep data collected in the summer of 2022 using a computer-vision algorithm for research purposes. Data were collected anonymously, and informed consent was obtained electronically. The institutional review board of the College of Staten Island approved the study (protocol #2022-0607). Participants were offered a respondent reward (raffle prize for 1 of 5 Nanit Ultimate Insights Subscription for 1 year). This study was conducted in accordance with the Declaration of Helsinki.

### Measures

Infant sleep was assessed objectively using Nanit camera monitors mounted over the infant’s crib at home. Using the accompanying app, users defined the area of the crib so that Nanit’s computer vision algorithm could translate motion-stillness patterns within the crib into sleep–wake patterns and within the defined parent pick-up area as parent^16^. Coding for nightly sleep metrics, Nanit is as accurate as and less intrusive than actigraphy and more accurate than parent report^17^.

This study included the following metrics: (1) total sleep time (TST; total minutes scored as sleep within the night sleep period); (2) minutes to sleep onset (MSO; latency until the first minute of 5 consecutive minutes of sleep after infants are placed in the crib); (3) sleep efficiency (SE; the proportion of time the infant spent asleep over the time the infant spent in the crib during the predefined nighttime sleep period) (4) number of night wakings (NW); and (5) number of parental visits to the crib (Visits).

### Data analysis plan

To investigate whether infants’ sleep was disrupted on or near the nights of the three heatwaves that occurred in the summer of 2022 (June 16–17, July 18–19, August 11–14)^18,19^, we performed a changepoint analysis on all sleep metrics. Changepoint analyses search for abrupt mean changes in times series data^20^. First, we calculated the within-subject mean of each sleep metric (TST, MSO, SE, NW, and Visits) over the 84 nights on which there was no heatwave. Next, we calculated the deviation for each sleep metric on each night compared to the within-subject mean. This deviation measure indicates how much better or worse a sleep metric is compared to the non-heatwave nights. For example, an infant with a parental visits deviation score of five on the heatwave night of July 18 was visited five more times during that night than their average parental visits during nights with average temperatures. Finally, we analyzed these deviations using changepoint analysis to identify changes within infants’ nightly sleep metrics compared to the within-subject mean. We analyzed the deviation from within-subject means to correct for the multilevel nature of the data. See Table S2 for average deviations per date of all sleep measures.

We used Wild Binary Segmentation^21^ as the changepoint with the R package WBS^22^ within R version 4.0.2^23^. WBS provides reliable location and number of changepoints estimates while remaining computationally efficient^24^. WBS computes the Cumulative Sums for random intervals in a sequence and subsequently tests the Cumulative Sum against a threshold to search for significant changepoints. A date is considered a significant changepoint if the corresponding Cumulative Sum exceeds the threshold, similar to how a difference is deemed significant if the p-value of a t-test is smaller than a set alpha value. See Table S3 for estimated threshold values and Median Absolute Devations per analysis. Using random intervals enabled us to search for mean shifts without making assumptions about the number of changepoints or the location of changepoints^21^. As suggested by Fryzlewicz^21^, we used a large *M* = 10,000 number of random draws and a threshold constant *C* = 1.

## Results

Deviations were found in all sleep metrics during the summer of 2022 in the UK. All dates referred to in this section refer to the night of that date.

### Total sleep time

**Figure 1:**
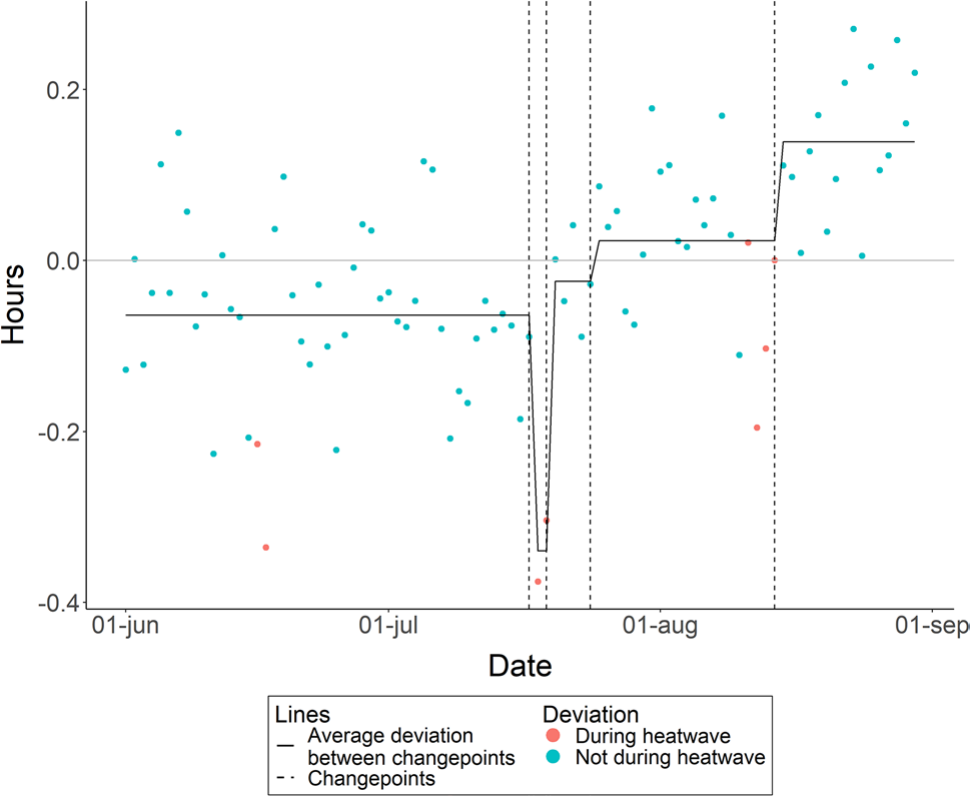
Within subject deviation from average sleep time over June, July, and August, 2022, excluding the 8 nights that met the definition of a heatwave. The horizontal line at zero represents the average sleep for all nights not included in a heat wave. A break in the line, marked by the vertical dashed line, indicates a significant changepoint in the deviation from the average.

The cumulative sum of infants’ total sleep time deviations exceeded the threshold on four occasions: July 17th, July 19th, July 24th, and August 14th (see Table S4 for Cumulative Sums of all dates). This indicates that infants’ total sleep time significantly changed during those nights (as depicted in Fig. 1). Up to and including July 17th infants slept on average 0.06 h less than the average of non-heatwave nights. During the second heatwave on July 18th and July 19th infants slept 0.34 h less than the average of non-heatwave nights. From July 20th to July 24th infants slept 0.02 h less than the average of non-heatwave nights. From July 25th until August 14th, the last day of the third heatwave, infants slept 0.02 h more than the average of non-heatwave nights. After the third heatwave ended on August 15th, infants slept 0.14 h more than the average of non-heatwave nights.

### Minutes to sleep onset

**Figure 2:**
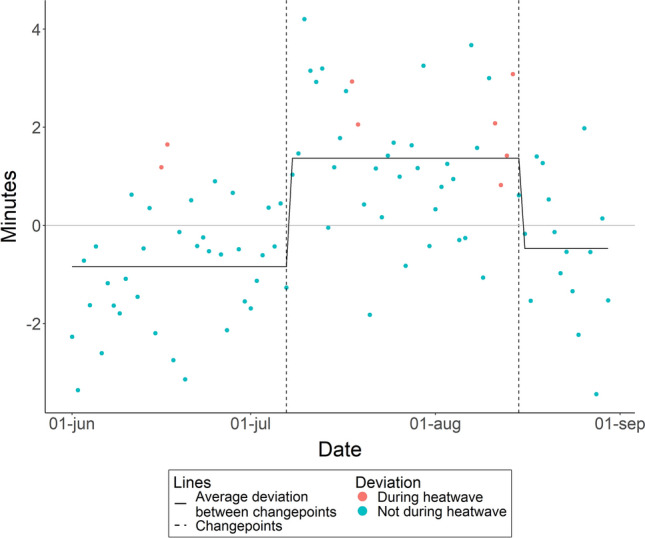
Within subject deviation from average minutes to sleep onset over June, July, and August, 2022, excluding the 8 nights that met the definition of a heatwave. The horizontal line at zero represents the average sleep for all nights not included in a heat wave. A break in the line, marked by the vertical dashed line, indicates a significant changepoint in the deviation from the average.

Infants sleep onset deviations significantly changed on two occasions: July 7th and August 15th (see Fig. 2 for a plot and Table S4 for Cumulative Sums of all dates). Up to and including July 7th infants fell asleep on average 0.84 min earlier than the average of non-heatwave nights. From July 8th to August 15th infants fell asleep 1.37 min later than the average of non-heatwave nights. After August 16th infants fell asleep 0.47 min earlier than the average of non-heatwave nights.

### Sleep efficiency

**Figure 3:**
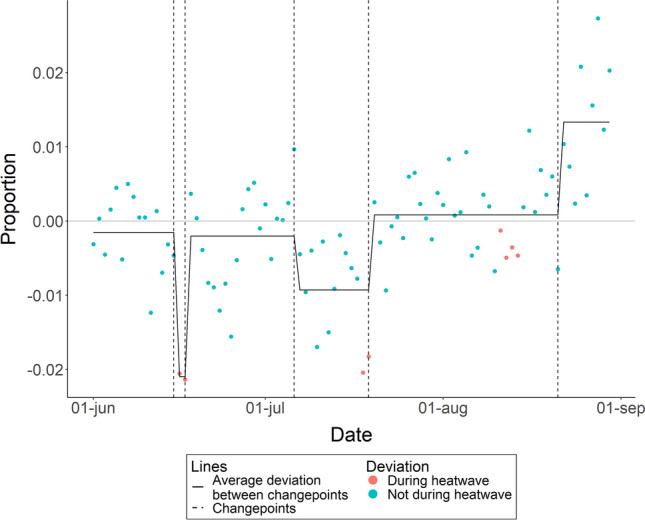
Within subject deviation from average sleep efficiency over June, July, and August, 2022, excluding the 8 nights that met the definition of a heatwave. The horizontal line at zero represents the average sleep for all nights not included in a heat wave. A break in the line, marked by the vertical dashed line, indicates a significant changepoint in the deviation from the average.

Deviations in infants’ sleep efficiency significantly changed on five occasions: June 15th, June 17th, July 6th, July 19th and August 21st (see Fig. 3 for a plot and Table S4 for Cumulative Sums of all dates). Up to and including June 15th infants’ sleep quality was on average 0.20% lower than the average of non-heatwave nights. During the first heatwave from June 16th to June 17th infants’ sleep quality was 2.1% lower. From June 18th to July 6th infants’ sleep quality was 0.20% lower. From July 7th until July 19th, the last day of the second heatwave, infants’ sleep quality was 0.90% lower. From July 20th up to and including August 21st infants’ sleep quality was 0.10% higher. From August 22nd infants’ sleep quality was 1.3% higher than the average of non-heatwave nights.

### Number of night wakings

**Figure 4:**
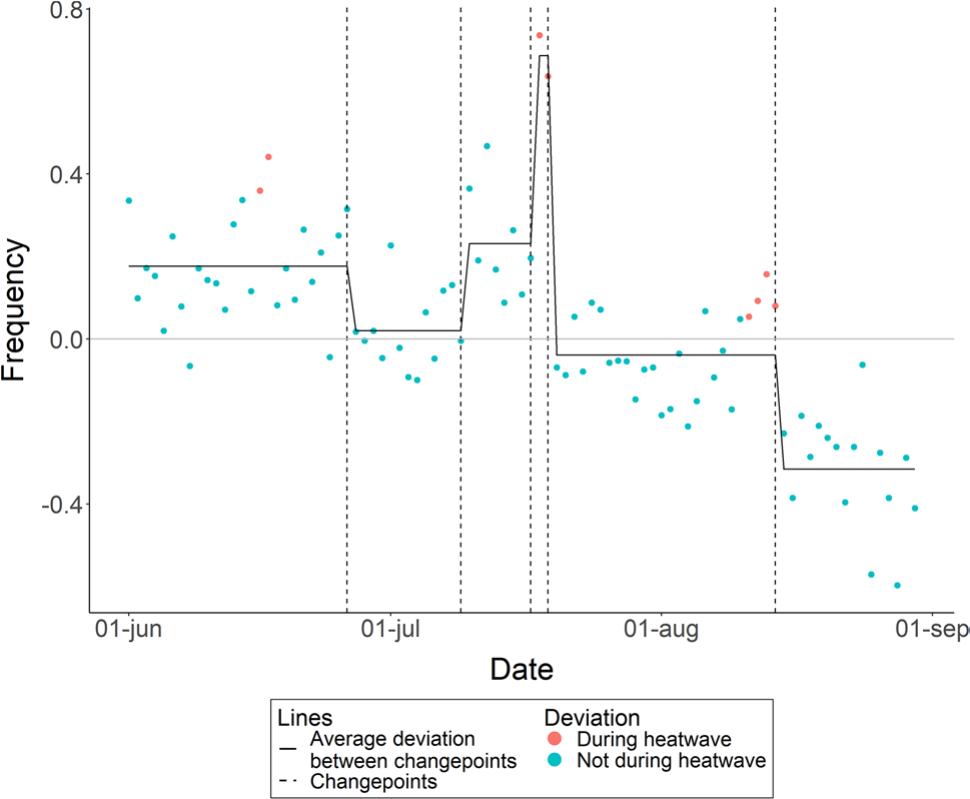
Within subject deviation from average number of nightly wakings over June, July, and August, 2022, excluding the 8 nights that met the definition of a heatwave. The horizontal line at zero represents the average sleep for all nights not included in a heat wave. A break in the line, marked by the vertical dashed line, indicates a significant changepoint in the deviation from the average.

Deviations in number of infants’ night wakings significantly changed on five occasions: June 26th, July 9th, July 17th, July 19th and August 14th (see Fig. 4 for a plot and Table S4 for Cumulative Sums of all dates). Up to and including June 26th infants woke up on average 0.18 times more than the average of non-heatwave nights. From June 27th to July 9th infants woke up 0.02 times more. From July 10th to July 17th infants woke up 0.23 times more than the average of non-heatwave nights. During the second heatwave on July 18th and July 19th infants woke up 0.69 times more. From July 20th up to and including the last day of the heatwave on August 14th infants woke up 0.04 times less than the average of non-heatwave nights. From August 15th infants woke up 0.32 times less.

### Number of parental visits to the crib

**Figure 5:**
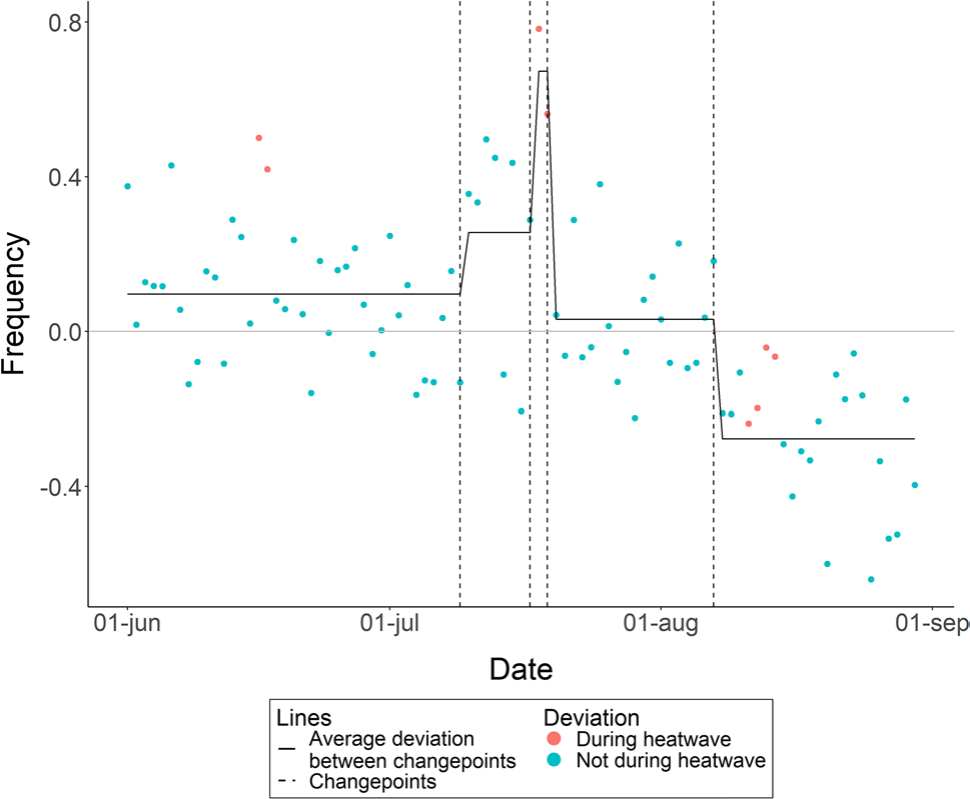
Within subject deviation from average number of parent visits over June, July, and August, 2022, excluding the 8 nights that met the definition of a heatwave. The horizontal line at zero represents the average sleep for all nights not included in a heat wave. A break in the line, marked by the vertical dashed line, indicates a significant changepoint in the deviation from the average.

The deviation in frequency of parental visits significantly changed on four occasions: on July 9th, July 17th, July 19th and August 7th (see Fig. 5 for a plot and Table S4 for Cumulative Sums of all dates). Up to and including July 9th parents intervened on average 0.10 times more than on non-heatwave nights. From July 10th to July 17th parents intervened 0.26 times more. During the second heatwave on July 18th and July 19th parents intervened 0.67 times more. From July 20th to August 7th parents intervened 0.03 times more. From August 8th parents intervened 0.28 times less.

## Discussion

This study examined the relation between temperature during the summer of 2022 and quality of infants’ sleep in the United Kingdom. The timing of sleep disruptions over the course of three heatwaves was associated with the absolute value of the temperature, across multiple sleep metrics, not the relative value that defines a heat wave. For the most part, sleep was not impacted in June when temperatures stayed below 88°, despite meeting the definition of a heat wave, but were significantly negatively impacted in July when temperatures reached over 100°.

More specifically, during the July heatwave when temperatures ranged from 96.8° to 102.2°, infants had less TST, took longer to fall asleep, had more fragmented sleep, and their parents visited them more frequently during the night, as compared to non-heatwave nights. During both the June and July heatwaves, infants had less efficient sleep compared to non-heatwave nights. After the August heatwave, all sleep metrics rebounded to better than average as compared to non-heatwave nights, suggesting that infants compensated for their disrupted sleep by sleeping more and with fewer interruptions especially once the temperature dropped below 85°.

In practical terms, the decrease in TST during the second heatwave meant that infants slept on average about 20 min less than usual for two nights in a row in addition to the few minutes of sleep they already had lost leading up to that heatwave. Studies that experimentally manipulate sleep deprivation in infants and school-aged children have shown that even small amounts of sleep deprivation over short periods can have detrimental effects. For example, in two small studies of healthy infants younger than 6 months old, missing a single morning nap or delaying a nap by just 2 h increased the frequency of obstructed sleep apnea during subsequent sleep^25,26^. In school-aged children, a week of mild sleep deprivation (about 39 min less sleep each night) resulted in a host of negative outcomes, including to their physical and psychological well-being^27^. We can extrapolate from these findings that ongoing sleep deprivation, which may increase as a result of increasingly unpredictable extreme weather patterns, has the potential for cascading negative effects on infant development. We must be especially mindful of the risks to infants with respiratory disorders and other health issues as the infants in these previous studies were all healthy, yet still experienced immediate respiratory changes after a short-term disruption to sleep.

Studies of infants’ recovery from sleep disruption provide further context for the interpretation of infants’ sleep patterns in this study. A recent study showed that after the change from daylight saving time to standard time, it took 4- to 24-month-olds at least a week to adjust to the new time, with their sleep midpoint shifting an average of 10 min each day of the week that sleep was measured^28^. In a small study of neonates who were awakened from REM or NREM sleep, total sleep time increased during recovery sleep^29^. Of course, setting the clocks back one hour or waking a sleeping infant once is not directly comparable to an ongoing loss of some amount of nightly sleep, but these findings are useful for understanding the recovery process from changes to infants’ sleep more generally. In the current study, infants’ TST, SE, and NW showed significant change points following the second heat wave that reflected recovery from the disruption to sleep. These sleep metrics also had another significant change point around the third heat wave that appears to reflect continued recovery and a rebound effect that continued to persist for over a week later. We did not anticipate such a protracted recovery period as there is very little work on recovery time in response to sleep disruption in healthy human infants. Future research will need to investigate the relation between the extent of sleep disruption and extent of recovery more systematically.

A visualization of the data shows that nightly changes in sleep closely track the changes in temperature. Even when the deviation from the average is not large enough to prompt a statistically significant change point, changes in sleep still peak on the nights with the highest temperatures. Taken together, the set of sleep metrics suggests that SE, as a composite measure that captures both total sleep time and fragmentation, may be more sensitive to sleep disruptions than the other sleep measures. Changes in other sleep measures may not cross a threshold, but the individual change points mirror each other from metric to metric.

Increased instances of disrupted sleep in infancy and early childhood have important implications for psychological health and development. For example, the quality of newly walking infants’ sleep the night before they learned to solve a novel problem predicted their readiness to learn^30^. Of the infants who ultimately solved the task, the more wake episodes and less efficient their sleep the night prior, the greater their difficulty in solving the problem the next day. These sleep metrics were negatively affected by the extreme temperatures that occurred during the July 2022 heat wave. Moreover, napping alone is insufficient for learning and memory consolidation in infancy^31^. There seems to be a unique role for night sleep in infant learning that cannot be substituted by shorter daytime sleep (naps). This may be because longer sleep episodes, characteristic of nighttime sleep, give infants time to cycle through REM and NREM states multiple times, both of which affect infant learning^32,33^. According to the synaptic homeostasis hypothesis, the primary function of night sleep is to reset synaptic strength after a day of learning to prepare for the next day’s learning^34^. Thus, climate disruptions that create occasional or ongoing sleep disruptions can leave infants vulnerable and unprepared for learning.

A hallmark accomplishment of infancy is the maturation of sleep, as reflected by a decrease in fragmentation, the establishment of a circadian rhythm, and, ultimately, sleeping through the night. Unpredictable interruptions to this process could potentially impact the establishment of mature biological functioning. This, in turn, could have cascading effects on later development. For example, sleep fragmentation at 10 months old was related to poor performance on a standardized assessment of cognitive development^35^. Similarly, total sleep time at 18 months old was related to executive functioning at 2 years old^36^. Likewise, higher sleep efficiency at 1 year of age predicted executive functioning (but not general cognition) at 4 years^37^. Again, these sleep metrics were susceptible to disruption during the 2022 heat wave.

A strength of this study is the ease with which objective metrics of multiple dimensions of sleep were collected for such a large dataset. Objective sleep assessments are crucial for thorough and accurate documentation, especially for behaviors that could easily be missed by parents in a self-report, such as when the infant can self-soothe after a night waking^38^. This is important in cases like this one where changes in behavior may be small, but meaningful and cumulative. A limitation of the study is that the maximum daytime temperature and not ambient room temperature was used, so we cannot be certain how close we were to capturing infants’ actual experiences. Nor did we capture infants’ daytime sleep (napping). Nevertheless, the finding that outdoor temperature was still sufficiently robust to predict patterns of night sleep suggests that the relation between climate change and infants’ sleep may be even more robust than what we were able to capture here. Another limitation is that the sample included only families who already owned the Nanit home video baby monitor. This could be a self-selecting group who could afford to purchase such a device (we have no way of knowing whether any were received as gifts). Given that global warming has had a disproportionate effect on residents of lower incomes^8^, future work must consider existing sleep health disparities in early childhood and emphasize that climate change will likely exacerbate pre-existing disparities.

This exploratory study raises several questions that are beyond the scope of this project. First, to directly test the hypotheses that sleep disruptions in infancy could negatively impact learning, future studies should conduct prospective behavioral studies of problem solving or memory consolidation before, during, and after extreme temperature events. Furthermore, future research would need to control for individual differences, such as age of the infant, duration of exposure to a heat wave, etc., to gain purchase on which factors put children at greatest risk for long-term repercussions and to what extent. Finally, because infants’ sleep rebounded, it is unlikely that infants’ sleep health met the definitions of disordered sleep^39^, but one question that remains unanswered is whether prolonged and ever-rising heat will lead to long-term accommodation or disruption of sleep health in infancy.

In sum, despite the importance of sleep for healthy learning and development in infancy, it remains relatively unstudied in the context of climate change. Models based on adult samples demonstrate that temperature increases due to climate change already impair sleep and predict up to 2 weeks of yearly sleep loss if temperature increases are not stabilized^8^. Moreover, the increased risk for the most vulnerable adult populations means that there is a strong likelihood that these patterns would be generalizable to infants, the most vulnerable among us, as well.

## Supplementary Information

Supplementary Information.

## Data Availability

Data is available in Supplementary Tables 2 and 4 and upon request to ST.
