# Author Correction: The impact of extreme summer temperatures in the United Kingdom on infant sleep: implications for learning and development

**DOI:** 10.1038/s41598-023-39008-6

**Published:** 2023-07-20

**Authors:** Sarah E. Berger, Monica R. Ordway, Emiel Schoneveld, Maristella Lucchini, Shambhavi Thakur, Thomas Anders, Liza Natale, Natalie Barnett

**Affiliations:** 1 College of Staten Island and the Graduate Center of the City University New York, 2800 Victory Blvd., 4S-108, Staten Island, NY 10314 USA; 2 Yale School of Nursing, Orange, USA; 3 Yale School of Medicine, West Haven, USA; 4 Research Institute of Child Development and Education, University of Amsterdam, Amsterdam, Netherlands; 5 Nanit Lab, New York, USA; 6 Brown University, Providence, USA; 7 New York University Grossman School of Medicine, New York, USA

Correction to: *Scientific Reports* 10.1038/s41598-023-37111-2, published online 21 June 2023

In the original version of this Article Emiel Schoneveld was incorrectly affiliated with ‘College of Staten Island and the Graduate Center of the City University New York, 2800 Victory Blvd., 4S-108, Staten Island, NY, 10314, USA’, ‘Yale School of Nursing, Orange, USA’, ‘Yale School of Medicine, West Haven, USA’, ‘Nanit Lab, New York, USA’, ‘Brown University, Providence, USA’ and ‘New York University Grossman School of Medicine, New York, USA’. The correct affiliation is listed below:

Research Institute of Child Development and Education, University of Amsterdam, Amsterdam, Netherlands.

As a result, the Affiliations have been renumbered.

The original Article has been corrected.

