# Supplementary material for: The impact of extreme summer temperatures in the United Kingdom on infant sleep: Implications for learning and development
Source: Sci Rep. 2023 Jun 21;13:10061. doi: 10.1038/s41598-023-37111-2 (PMC10284886; doi:10.1038/s41598-023-37111-2)
Supplement: Supplementary file 1 — Supplementary Information. [file 41598_2023_37111_MOESM1_ESM.docx]

**Supplementary materials**

**Figure S1**

**
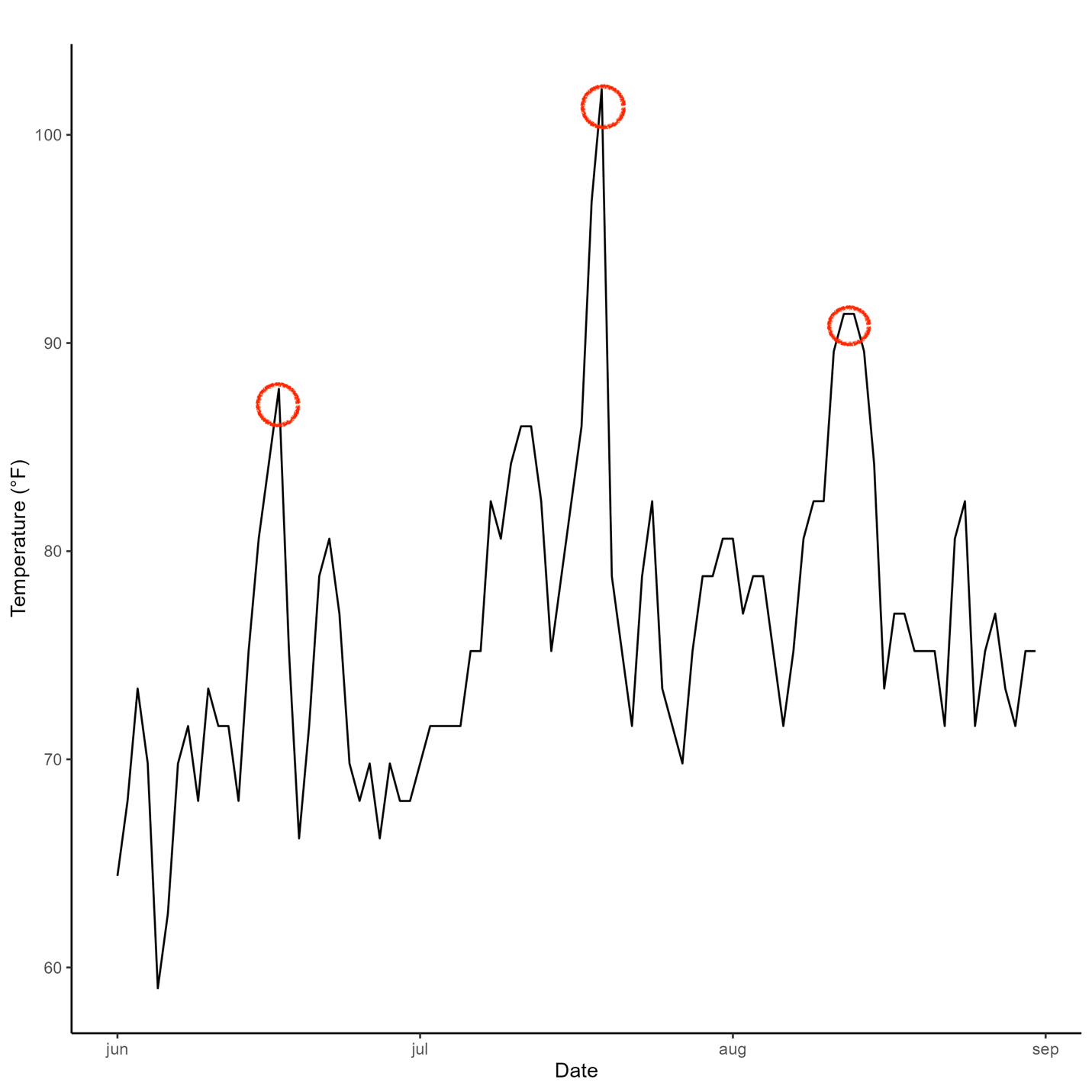
**

Note: Historical weather data for London, England, for the summer of 2022.^40^ Nights of heat waves circled in red.

**Table S2**

*Average deviations per night for the sleep measures: total sleep time in hours, minutes to sleep onset, sleep efficiency, number of night wakings and number of parental visits*

| Date | Total sleep time | Sleep onset | Sleep efficiency | Night wakings | Parental  visits |
| --- | --- | --- | --- | --- | --- |
| 2022-06-01 | -0.128 | -2.269 | -0.003 | 0.336 | 0.376 |
| 2022-06-02 | 0.001 | -3.357 | 0.000 | 0.099 | 0.017 |
| 2022-06-03 | -0.122 | -0.719 | -0.005 | 0.172 | 0.127 |
| 2022-06-04 | -0.038 | -1.626 | 0.002 | 0.153 | 0.118 |
| 2022-06-05 | 0.113 | -0.429 | 0.004 | 0.020 | 0.117 |
| 2022-06-06 | -0.038 | -2.606 | -0.005 | 0.249 | 0.429 |
| 2022-06-07 | 0.150 | -1.180 | 0.005 | 0.079 | 0.056 |
| 2022-06-08 | 0.057 | -1.633 | 0.003 | -0.065 | -0.137 |
| 2022-06-09 | -0.077 | -1.795 | 0.000 | 0.171 | -0.079 |
| 2022-06-10 | -0.040 | -1.089 | 0.001 | 0.143 | 0.156 |
| 2022-06-11 | -0.226 | 0.628 | -0.012 | 0.135 | 0.139 |
| 2022-06-12 | 0.006 | -1.456 | 0.001 | 0.071 | -0.083 |
| 2022-06-13 | -0.057 | -0.469 | -0.007 | 0.278 | 0.289 |
| 2022-06-14 | -0.066 | 0.354 | -0.003 | 0.337 | 0.244 |
| 2022-06-15 | -0.207 | -2.198 | -0.005 | 0.116 | 0.020 |
| 2022-06-16 | -0.215 | 1.183 | -0.021 | 0.360 | 0.500 |
| 2022-06-17 | -0.336 | 1.650 | -0.021 | 0.441 | 0.420 |
| 2022-06-18 | 0.037 | -2.747 | 0.004 | 0.082 | 0.079 |
| 2022-06-19 | 0.098 | -0.135 | 0.000 | 0.171 | 0.058 |
| 2022-06-20 | -0.041 | -3.137 | -0.004 | 0.095 | 0.237 |
| 2022-06-21 | -0.095 | 0.514 | -0.008 | 0.265 | 0.045 |
| 2022-06-22 | -0.122 | -0.419 | -0.009 | 0.138 | -0.159 |
| 2022-06-23 | -0.029 | -0.245 | -0.012 | 0.210 | 0.182 |
| 2022-06-24 | -0.101 | -0.527 | -0.008 | -0.044 | -0.003 |
| 2022-06-25 | -0.222 | 0.901 | -0.016 | 0.251 | 0.159 |
| 2022-06-26 | -0.087 | -0.590 | -0.005 | 0.315 | 0.168 |
| 2022-06-27 | -0.009 | -2.136 | 0.002 | 0.018 | 0.216 |
| 2022-06-28 | 0.042 | 0.665 | 0.004 | -0.004 | 0.069 |
| 2022-06-29 | 0.035 | -0.487 | 0.005 | 0.020 | -0.059 |
| 2022-06-30 | -0.045 | -1.548 | -0.001 | -0.046 | 0.003 |
| 2022-07-01 | -0.037 | -1.692 | 0.002 | 0.227 | 0.247 |
| 2022-07-02 | -0.072 | -1.129 | -0.005 | -0.021 | 0.042 |
| 2022-07-03 | -0.078 | -0.608 | 0.000 | -0.092 | 0.120 |
| 2022-07-04 | -0.048 | 0.362 | 0.000 | -0.099 | -0.163 |
| 2022-07-05 | 0.116 | -0.427 | 0.002 | 0.065 | -0.126 |
| 2022-07-06 | 0.106 | 0.447 | 0.010 | -0.047 | -0.131 |
| 2022-07-07 | -0.080 | -1.268 | -0.004 | 0.118 | 0.035 |
| 2022-07-08 | -0.208 | 1.034 | -0.010 | 0.131 | 0.157 |
| 2022-07-09 | -0.153 | 1.466 | -0.004 | -0.004 | -0.132 |
| 2022-07-10 | -0.167 | 4.203 | -0.017 | 0.365 | 0.356 |
| 2022-07-11 | -0.092 | 3.152 | -0.003 | 0.191 | 0.334 |
| 2022-07-12 | -0.048 | 2.924 | -0.015 | 0.468 | 0.497 |
| 2022-07-13 | -0.081 | 3.198 | -0.009 | 0.168 | 0.449 |
| 2022-07-14 | -0.063 | -0.044 | -0.002 | 0.088 | -0.112 |
| 2022-07-15 | -0.076 | 1.185 | -0.004 | 0.264 | 0.436 |
| 2022-07-16 | -0.185 | 1.778 | -0.006 | 0.108 | -0.205 |
| 2022-07-17 | -0.089 | 2.736 | -0.008 | 0.196 | 0.288 |
| 2022-07-18 | -0.376 | 2.931 | -0.020 | 0.736 | 0.783 |
| 2022-07-19 | -0.304 | 2.058 | -0.018 | 0.637 | 0.563 |
| 2022-07-20 | 0.001 | 0.426 | 0.003 | -0.069 | 0.042 |
| 2022-07-21 | -0.048 | -1.822 | -0.003 | -0.087 | -0.063 |
| 2022-07-22 | 0.041 | 1.162 | -0.009 | 0.054 | 0.288 |
| 2022-07-23 | -0.089 | 0.167 | -0.001 | -0.079 | -0.067 |
| 2022-07-24 | -0.028 | 1.420 | 0.001 | 0.088 | -0.041 |
| 2022-07-25 | 0.087 | 1.685 | -0.002 | 0.071 | 0.381 |
| 2022-07-26 | 0.039 | 0.994 | 0.006 | -0.058 | 0.014 |
| 2022-07-27 | 0.058 | -0.825 | 0.006 | -0.053 | -0.130 |
| 2022-07-28 | -0.060 | 1.635 | 0.002 | -0.054 | -0.053 |
| 2022-07-29 | -0.075 | 1.169 | 0.000 | -0.147 | -0.223 |
| 2022-07-30 | 0.007 | 3.256 | -0.002 | -0.074 | 0.082 |
| 2022-07-31 | 0.178 | -0.421 | 0.004 | -0.069 | 0.142 |
| 2022-08-01 | 0.104 | 0.328 | 0.002 | -0.185 | 0.031 |
| 2022-08-02 | 0.111 | 0.784 | 0.008 | -0.170 | -0.081 |
| 2022-08-03 | 0.023 | 1.253 | 0.001 | -0.036 | 0.228 |
| 2022-08-04 | 0.016 | 0.943 | 0.001 | -0.212 | -0.095 |
| 2022-08-05 | 0.071 | -0.297 | 0.009 | -0.150 | -0.081 |
| 2022-08-06 | 0.041 | -0.258 | -0.005 | 0.067 | 0.036 |
| 2022-08-07 | 0.073 | 3.676 | -0.004 | -0.093 | 0.182 |
| 2022-08-08 | 0.169 | 1.581 | 0.004 | -0.028 | -0.211 |
| 2022-08-09 | 0.030 | -1.066 | 0.002 | -0.171 | -0.213 |
| 2022-08-10 | -0.111 | 3.002 | -0.007 | 0.049 | -0.106 |
| 2022-08-11 | 0.021 | 2.082 | -0.001 | 0.054 | -0.237 |
| 2022-08-12 | -0.195 | 0.821 | -0.005 | 0.093 | -0.198 |
| 2022-08-13 | -0.103 | 1.422 | -0.004 | 0.157 | -0.041 |
| 2022-08-14 | 0.000 | 3.085 | -0.005 | 0.080 | -0.065 |
| 2022-08-15 | 0.111 | 0.613 | 0.002 | -0.228 | -0.291 |
| 2022-08-16 | 0.098 | -0.173 | 0.012 | -0.385 | -0.425 |
| 2022-08-17 | 0.009 | -1.536 | 0.001 | -0.186 | -0.308 |
| 2022-08-18 | 0.128 | 1.407 | 0.007 | -0.286 | -0.332 |
| 2022-08-19 | 0.170 | 1.269 | 0.004 | -0.211 | -0.231 |
| 2022-08-20 | 0.034 | 0.527 | 0.006 | -0.240 | -0.600 |
| 2022-08-21 | 0.095 | -0.137 | -0.006 | -0.261 | -0.111 |
| 2022-08-22 | 0.208 | -0.975 | 0.010 | -0.396 | -0.175 |
| 2022-08-23 | 0.271 | -0.538 | 0.007 | -0.261 | -0.057 |
| 2022-08-24 | 0.005 | -1.342 | 0.002 | -0.062 | -0.165 |
| 2022-08-25 | 0.227 | -2.229 | 0.021 | -0.571 | -0.640 |
| 2022-08-26 | 0.106 | 1.980 | 0.003 | -0.276 | -0.334 |
| 2022-08-27 | 0.123 | -0.543 | 0.016 | -0.385 | -0.535 |
| 2022-08-28 | 0.258 | -3.440 | 0.027 | -0.597 | -0.524 |
| 2022-08-29 | 0.161 | 0.143 | 0.012 | -0.288 | -0.176 |
| 2022-08-30 | 0.220 | -1.528 | 0.020 | -0.410 | -0.395 |

**Table S3**

*Threshold and Median Absolute Deviation as estimated within the Wild Binary Segmentation analysis for each sleep measure*

| Sleep measures | Threshold | Median Absolute Deviation |
| --- | --- | --- |
| Total sleep time (hrs) | 0.363 | 0.093 |
| Sleep onset (mins) | 4.543 | 1.163 |
| Sleep efficiency (ppn) | 0.024 | 0.006 |
| Number night wakings | 0.493 | 0.126 |
| Number parental visits | 0.695 | 0.178 |

**Table S4**

*Cumulative Sums of all dates as estimated within the Wild Binary Segmentation analysis for each sleep measure*

| Date | Total sleep time | Sleep onset | Sleep efficiency | Number night wakings | Number parental interventions |
| --- | --- | --- | --- | --- | --- |
| 2022-06-01 | 0.092 | 0.769 | 0.002 | 0.220 | 0.253 |
| 2022-06-02 | 0.087 | 2.106 | 0.003 | 0.052 | 0.090 |
| 2022-06-03 | 0.059 | 0.607 | 0.006 | 0.013 | 0.008 |
| 2022-06-04 | 0.201 | 0.607 | 0.002 | 0.116 | 0.001 |
| 2022-06-05 | 0.107 | 1.539 | 0.007 | 0.162 | 0.299 |
| 2022-06-06 | 0.133 | 1.057 | 0.008 | 0.198 | 0.418 |
| 2022-06-07 | 0.065 | 0.436 | 0.001 | 0.102 | 0.134 |
| 2022-06-08 | 0.242 | 0.115 | 0.004 | 0.186 | 0.041 |
| 2022-06-09 | 0.027 | 0.436 | 0.000 | 0.026 | 0.255 |
| 2022-06-10 | 0.137 | 2.276 | 0.013 | 0.006 | 0.011 |
| 2022-06-11 | 0.164 | 1.142 | 0.010 | 0.068 | 0.188 |
| 2022-06-12 | 0.055 | 1.142 | 0.006 | 0.361 | 0.319 |
| 2022-06-13 | 0.007 | 0.582 | 0.003 | 0.042 | 0.032 |
| 2022-06-14 | 0.356 | 1.805 | 0.001 | 0.156 | 0.201 |
| 2022-06-15 | 0.005 | 3.639 | **0.026*** | 0.232 | 0.502 |
| 2022-06-16 | 0.102 | 0.330 | 0.001 | 0.057 | 0.057 |
| 2022-06-17 | 0.360 | 3.750 | **0.025*** | 0.336 | 0.559 |
| 2022-06-18 | 0.043 | 1.847 | 0.002 | 0.063 | 0.015 |
| 2022-06-19 | 0.208 | 2.122 | 0.005 | 0.054 | 0.136 |
| 2022-06-20 | 0.050 | 2.904 | 0.015 | 0.129 | 0.136 |
| 2022-06-21 | 0.019 | 0.789 | 0.000 | 0.090 | 0.245 |
| 2022-06-22 | 0.050 | 0.123 | 0.003 | 0.051 | 0.277 |
| 2022-06-23 | 0.051 | 0.159 | 0.003 | 0.215 | 0.131 |
| 2022-06-24 | 0.132 | 1.124 | 0.005 | 0.267 | 0.160 |
| 2022-06-25 | 0.095 | 1.055 | 0.007 | 0.045 | 0.006 |
| 2022-06-26 | 0.195 | 1.921 | 0.023 | **0.515*** | 0.043 |
| 2022-06-27 | 0.039 | 1.817 | 0.003 | 0.016 | 0.216 |
| 2022-06-28 | 0.005 | 0.814 | 0.001 | 0.017 | 0.090 |
| 2022-06-29 | 0.113 | 1.708 | 0.005 | 0.050 | 0.043 |
| 2022-06-30 | 0.005 | 0.102 | 0.002 | 0.206 | 0.225 |
| 2022-07-01 | 0.034 | 0.400 | 0.007 | 0.258 | 0.136 |
| 2022-07-02 | 0.005 | 1.833 | 0.005 | 0.061 | 0.055 |
| 2022-07-03 | 0.022 | 0.686 | 0.000 | 0.005 | 0.371 |
| 2022-07-04 | 0.228 | 0.558 | 0.002 | 0.131 | 0.028 |
| 2022-07-05 | 0.007 | 0.618 | 0.009 | 0.079 | 0.003 |
| 2022-07-06 | 0.304 | 1.212 | **0.027*** | 0.194 | 0.258 |
| 2022-07-07 | 0.083 | **9.672*** | 0.004 | 0.009 | 0.086 |
| 2022-07-08 | 0.039 | 0.305 | 0.004 | 0.105 | 0.204 |
| 2022-07-09 | 0.010 | 2.447 | 0.009 | **0.501*** | 0.727 |
| 2022-07-10 | 0.143 | 0.963 | 0.010 | 0.123 | 0.016 |
| 2022-07-11 | 0.031 | 0.161 | 0.009 | 0.196 | 0.128 |
| 2022-07-12 | 0.022 | 0.193 | 0.004 | 0.278 | 0.034 |
| 2022-07-13 | 0.013 | 4.523 | 0.009 | 0.057 | 0.477 |
| 2022-07-14 | 0.010 | 0.869 | 0.002 | 0.124 | 0.387 |
| 2022-07-15 | 0.106 | 2.093 | 0.004 | 0.110 | 0.493 |
| 2022-07-16 | 0.068 | 0.862 | 0.001 | 0.063 | 0.349 |
| 2022-07-17 | **0.383*** | 0.138 | 0.016 | **0.792*** | 0.781 |
| 2022-07-18 | 0.051 | 0.634 | 0.002 | 0.070 | 0.155 |
| 2022-07-19 | **0.377*** | 3.585 | **0.032*** | **1.021*** | 0.872 |
| 2022-07-20 | 0.034 | 1.590 | 0.004 | 0.013 | 0.074 |
| 2022-07-21 | 0.063 | 2.853 | 0.008 | 0.108 | 0.248 |
| 2022-07-22 | 0.092 | 0.703 | 0.008 | 0.094 | 0.279 |
| 2022-07-23 | 0.044 | 1.131 | 0.001 | 0.144 | 0.018 |
| 2022-07-24 | **0.691*** | 0.187 | 0.002 | 0.012 | 0.318 |
| 2022-07-25 | 0.034 | 0.488 | 0.012 | 0.247 | 0.428 |
| 2022-07-26 | 0.013 | 1.898 | 0.000 | 0.004 | 0.102 |
| 2022-07-27 | 0.141 | 1.819 | 0.007 | 0.001 | 0.055 |
| 2022-07-28 | 0.011 | 0.329 | 0.001 | 0.079 | 0.144 |
| 2022-07-29 | 0.061 | 2.248 | 0.003 | 0.061 | 0.295 |
| 2022-07-30 | 0.213 | 2.734 | 0.004 | 0.004 | 0.043 |
| 2022-07-31 | 0.057 | 0.530 | 0.001 | 0.117 | 0.078 |
| 2022-08-01 | 0.005 | 1.140 | 0.007 | 0.011 | 0.144 |
| 2022-08-02 | 0.123 | 0.332 | 0.006 | 0.115 | 0.218 |
| 2022-08-03 | 0.005 | 0.219 | 0.000 | 0.125 | 0.258 |
| 2022-08-04 | 0.047 | 1.392 | 0.007 | 0.044 | 0.010 |
| 2022-08-05 | 0.021 | 0.027 | 0.014 | 0.203 | 0.101 |
| 2022-08-06 | 0.022 | 3.228 | 0.001 | 0.142 | 0.198 |
| 2022-08-07 | 0.114 | 1.481 | 0.007 | 0.046 | 1.620 |
| 2022-08-08 | 0.099 | 3.016 | 0.001 | 0.101 | 0.002 |
| 2022-08-09 | 0.302 | 2.946 | 0.008 | 0.361 | 0.087 |
| 2022-08-10 | 0.093 | 0.651 | 0.004 | 0.004 | 0.092 |
| 2022-08-11 | 0.153 | 1.421 | 0.003 | 0.034 | 0.027 |
| 2022-08-12 | 0.065 | 0.425 | 0.001 | 0.079 | 0.167 |
| 2022-08-13 | 0.122 | 1.603 | 0.001 | 0.054 | 0.017 |
| 2022-08-14 | **0.423*** | 1.748 | 0.016 | **1.486*** | 0.388 |
| 2022-08-15 | 0.010 | **6.071*** | 0.007 | 0.111 | 0.095 |
| 2022-08-16 | 0.078 | 0.964 | 0.008 | 0.141 | 0.116 |
| 2022-08-17 | 0.114 | 2.346 | 0.004 | 0.071 | 0.017 |
| 2022-08-18 | 0.030 | 0.097 | 0.002 | 0.053 | 0.073 |
| 2022-08-19 | 0.097 | 0.662 | 0.002 | 0.033 | 0.268 |
| 2022-08-20 | 0.044 | 2.892 | 0.011 | 0.015 | 0.423 |
| 2022-08-21 | 0.185 | 0.593 | **0.046*** | 0.145 | 0.045 |
| 2022-08-22 | 0.045 | 0.309 | 0.002 | 0.095 | 0.083 |
| 2022-08-23 | 0.191 | 1.353 | 0.005 | 0.217 | 0.076 |
| 2022-08-24 | 0.164 | 0.627 | 0.013 | 0.360 | 0.539 |
| 2022-08-25 | 0.092 | 3.074 | 0.012 | 0.209 | 0.216 |
| 2022-08-26 | 0.012 | 1.784 | 0.009 | 0.078 | 0.160 |
| 2022-08-27 | 0.117 | 3.395 | 0.016 | 0.218 | 0.007 |
| 2022-08-28 | 0.069 | 2.534 | 0.011 | 0.219 | 0.298 |
| 2022-08-29 | 0.042 | 1.182 | 0.006 | 0.086 | 0.155 |
| 2022-08-30 | Not estimated | Not estimated | Not estimated | Not estimated | Not estimated |

** Significantly greater than corresponding threshold value (th_totalsleeptime_ = 0.363, th_sleeponset_ = 4.543, th_sleepefficiency_ = 0.024, th_nightwakings_ = 0.493 and th_parentalinterventions_ = 0.695*

*Note. ^a^ No Cumulative Sums for the last date were estimated since no data after the 30^th^ of August was present.*
